# Making Medicines Baby Size: The Challenges in Bridging the Formulation Gap in Neonatal Medicine

**DOI:** 10.3390/ijms20112688

**Published:** 2019-05-31

**Authors:** Fiona O’Brien, David Clapham, Kamelia Krysiak, Hannah Batchelor, Peter Field, Grazia Caivano, Marisa Pertile, Anthony Nunn, Catherine Tuleu

**Affiliations:** 1 School of Pharmacy, Royal College of Surgeons in Ireland, 111 St Stephens Green Dublin 2, Ireland; fionaobrien@rcsi.ie (F.O.); kameliakrysiak@rcsi.ie (K.K.); 2 14 Tailors, Bishops Stortford CM23 4FQ, UK; david.clapham@ntlworld.com; 3 College of Medical and Dental Sciences, Institute of Clinical Sciences, University of Birmingham, Birmingham B15 2TT, UK; H.K.Batchelor@bham.ac.uk; 4 University College London School of Pharmacy, 29-39 Brunswick Square, London WC1N 1AX, UK; peter.field@ucl.ac.uk; 5 Chiesi Farmaceutici S.p.A. Largo Francesco Belloli 11/A—43122 Parma, Italy; g.caivano@chiesi.com (G.C.); m.pertile@chiesi.com (M.P.); 6 Department of Women’s and Children’s Health, University of Liverpool, Liverpool Women’s Hospital, Liverpool L8 7SS, UK; a.j.nunn@liverpool.ac.uk

**Keywords:** neonates, formulation, product development, formulation development, oral, parenteral, topical, inhaled, intra nasal, biopharmaceutics, administration, excipient, NICU, device, medication error, dosage form

## Abstract

The development of age-appropriate formulations should focus on dosage forms that can deliver variable yet accurate doses that are safe and acceptable to the child, are matched to his/her development and ability, and avoid medication errors. However, in the past decade, the medication needs of neonates have largely been neglected. The aim of this review is to expand on what differentiates the needs of preterm and term neonates from those of the older paediatric subsets, in terms of environment of care, ability to measure and administer the dose (from the perspective of the patient and carer, the routes of administration, the device and the product), neonatal biopharmaceutics and regulatory challenges. This review offers insight into those challenges posed by the formulation of medicinal products for neonatal patients in order to support the development of clinically relevant products.

## 1. Introduction

Neonates are not small adults, and neither can they be classified as small children when it comes to medicinal products and their formulation development.

Neonates include term, post-term and preterm babies. The neonatal period for term and post-term newborn infants is defined as the day of birth plus 27 days. The neonatal period for preterm newborn infants is defined as the day of birth through to the expected date of delivery plus 27 days [1].

Each year, some 15 million babies arrive in the world, more than one in 10 babies are born prematurely, according to the report ‘Born Too Soon: The Global Action Report on Preterm Birth’ (2012) [2]. Even if born at term and ready to grow outside of the mother’s womb, most organs and their functions are still immature. This immaturity of organ and function is more profound and impactful in preterm infants. For example, neonates have reduced gastric emptying, intestinal transit time and surface area, and transporter immaturity, which have relevance for oral drug delivery. Additionally, the skin barrier may not be fully formed, and respiratory function may be immature. A host of other physiological factors such as gastro intestinal (GI) pH, body surface to volume ratio, body fat to lean tissue ratio are also different and are known to change rapidly with time [3].

The underlying complexity of pharmacology and biopharmaceutics in neonates, especially in preterm babies, can lead to altered and variable pharmacokinetics and pharmacodynamics even compared to that in young babies [4,5,6]. In turn, this can lead to potential lack of efficacy or reduced safety of a medicinal product leading to additional special requirements for development of products for these age groups. This includes (amongst others) formulation aspects, dosage form choice, drug administration considerations and storage and handling advice.

The recent European Medicines Agency (EMA) report to the European Commission on the experience acquired as a result of the first 10 years application of the Paediatric Regulation, acknowledged that neonates still represent a particularly neglected paediatric subpopulation in the development of medicines despite the regulatory push [7].

In paediatric patients, there is still significant off-label use of medicines due to a lack of medicines developed and authorised for the specific needs of the very young [8,9]. This remains an even more significant problem in the neonatal population, due to the difficulty in conducting the necessary clinical trials in vulnerable subsets with lower patient numbers. Considering the extra challenges they present, pharmaceutical companies are not incentivised to formulate medicines for the neonatal population. In fact, trials open for recruiting neonates were included in only a quarter of all agreed paediatric investigation plans (PIPs), often at the request of the EMA Paediatric Committee (PDCO), due to an array of reasons: Lack of neonate specific indications, recruitment/enrollment challenges, lack of incentives [10]. There is a ‘Catch 22′ situation because to protect neonates, trials have been deferred so that safety and efficacy data are obtained in older age groups meanwhile the unmet needs gap widens, necessitating off-label use [11]. An international consortium of experts has produced a white paper to facilitate successful neonatal clinical trials of medicines and includes useful information on neonatal dosage forms and formulations [12].

The situation is not helped by a relative lack of relevant guidelines on the development of medicines for neonates (let alone those born prematurely). In terms of international guidance, it is only in the latest revision of the ICH E11 Guideline on Clinical Investigation of Medicinal Products in the Paediatric Population (2018) [1] that neonates are specifically mentioned as an age classification and paediatric subgroup. In these guidelines, a rather general and brief set of considerations are made in terms of formulation and around polypharmacy via parenteral routes of administration in the hospital setting. The capability to administer small volumes in relation to dosing error is also mentioned. This is insufficient considering the much wider and complex needs of neonates [13] and lack of clarity about how this limited guidance is to be translated into patient-centric product development, which meets with regulatory approval.

The common ground in current guidelines is that there are pointers to the lack of safety data on excipients and that available data generally does not apply to neonates requiring further justification including provision of non-clinical safety data [14,15].

The aim of this review is, therefore, to provide insights and factors to consider in order to assist those developing products for neonates, but with little or no neonatal medicine knowledge or paediatric formulation development background, to overcome the range of challenges posed by this patient group and so enhance the provision of clinically relevant products. This includes factors that differentiate the needs of preterm and term neonates from those of the older paediatric subsets, in terms of environment of care, ability to measure and administer the dose (from the patient, the routes of administration, the device and the product perspectives), formulation development, neonatal biopharmaceutics and regulatory challenges.

## 2. Formulation Considerations

### 2.1. Environment of Care

Neonates who receive treatment in hospital, will most often be located in the Neonatal Intensive Care Unit (NICU). The NICU admits high-risk premature and full-term neonates with serious medical or surgical conditions.

Factors which may necessitate neonatal admission include (modified from [16])

- Premature birth <37 weeks gestation
- Delayed birth >42 weeks gestation
- Birth weight <2500 g
- Concern about size, e.g., intrauterine growth restriction (IUGR)
- Medication or resuscitation required in the delivery room
- Birth defects, e.g., congenital heart defects, intraventricular haemorrhage, macrosomia, retinopathy of prematurity (ROP)
- Respiratory problems including RDS (respiratory distress syndrome) and BDP (bronchopulmonary dysplasia)
- Infection (including neonatal sepsis)
- Seizures
- Hypoglycemia
- Requiring additional support (extra oxygen or monitoring, body temperature control support, intravenous (IV) therapy, or medications) or specialized treatments (blood transfusion)
- Feeding issues
- Jaundice

Often multiple conditions exist concomitantly leading to a need for polypharmacy.

**Figure 1:**
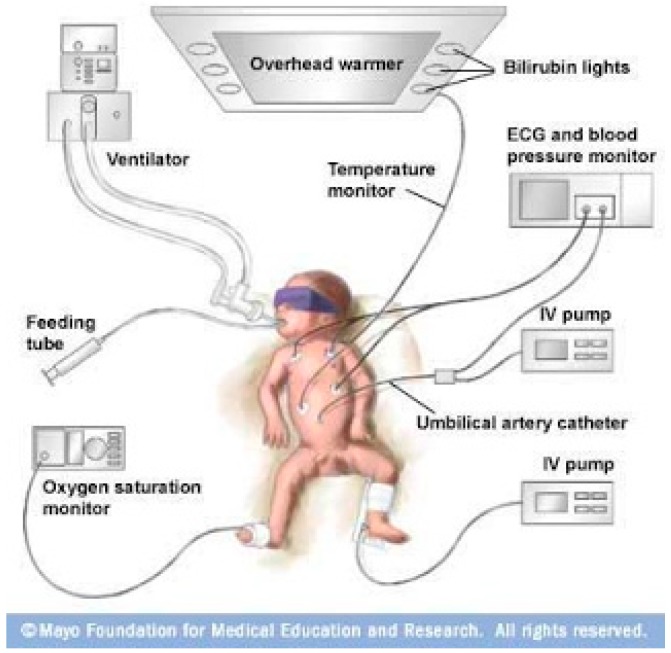
Schematic of NICU support for neonate (used with permission of Mayo Foundation for Medical Education and Research, all rights reserved).

Neonates admitted to the NICU often require periods in specialised incubators to maintain optimum environmental conditions and may also be attached to electrocardiogram (ECG), oxygen saturation and blood pressure monitors (Figure 1). In addition, they may require respiratory support through oxygen supplementation or require phototherapy for jaundice [17]. The majority of pharmaceutical medicinal products administered to neonates are liquid oral or parenteral formulations [18]. In this population, the environment of care is critical with the vast majority of IV infusions delivered in an intensive care setting where the additional environmental requirements of light, temperature and oxygen may impact the physicochemical stability of medication being delivered [19]. The effect of the environment of care should be a critical consideration in the formulation design of a parenteral product intended for this patient population and in use stability studies will be discussed in more detail below.

Neonates in incubators also provide some challenges for the provision of suitable nutrition and hydration requiring enteral or parenteral feeding. Given the limited number of access points and the requirement for polypharmacy that is common in this patient group, there are more opportunities for stability challenges, interactions and incompatibilities.

### 2.2. Ability to Dose: Patient (Developmental Age)/Physiological/Administration Routes Factors to Consider

Some drugs can be administered to these patient groups orally, topically, via inhalation or indeed by any of the usual administration routes and the specific issues associated with the ability to dose via these routes is discussed under each of these sections below. However, the main route of administration in the neonatal patient is parenteral and in particular the IV route for seriously ill preterm and term neonates [20].

#### 2.2.1. Parenteral Delivery

The neonatal IV infusion is a demanding process, involving vulnerable patients and complex IV administration apparatus. Venous access in neonates can be via a catheter with tip placed in the vena cava, a central venous catheter (CVC) (threaded through from a peripheral vein and known as a ‘PICC’ or peripherally-inserted central catheter, inserted through the umbilical vein (UVC) or placed surgically) or via a peripheral cannula or catheter (accessing smaller veins in the hands, arms and feet). A UVC remains in situ for up to two weeks after birth and PICC may last for several weeks whilst cannulas and catheters accessing the small peripheral veins may only be patent for hours or days [21].

All cannulas and catheters require scrupulous care to avoid blockage and infection. Since blood flow in central versus peripheral veins is greater, an administered drug is rapidly diluted [22]. Some irritant (chemotherapy, amiodarone, vasopressors [22] and hyperosmolar (glucose >12.5%, total parenteral nutrition (TPN)) [23] medications, may be indicated for central administration only. Many medications are compatible with a peripheral route of administration. However, some may cause phlebitis (e.g., dopamine, dobutamine, sodium bicarbonate, calcium gluconate) [24].

The choice of available catheters is summarised in Table 1 alongside characteristics and issues associated with each.

Tubing of small internal diameter is often used to reduce the dead space in apparatus for IV drug administration. Even so, drugs may be exposed to adverse temperatures and light from the point of administration until they reach the vascular system. This should be taken into account when assessing in-use stability as well as pharmacokinetics and clinical outcomes.

An important formulation consideration associated with IV administration in preterm and term neonates is the volume of fluid that can be tolerated. Neonates, especially those delivered preterm, will have critical fluid and electrolyte requirements. At full term, a neonate has a fluid allowance of 100–140 mL/kg/day resulting in administration of 10–20 mL/hr, which includes all routes of administration [27]. In the case of severely fluid restricted neonates, IV fluids and drugs must be concentrated and so may need to be infused at flow rates as low as 0.02 mL/hr. Formulations for IV drugs and infusions should be designed with such requirements in mind and should take account of the sickest babies requiring multiple therapies, each with their own method of administration to be considered.

Generally, drugs administered intravenously should not be required to be administered in a fixed volume. It is preferable to investigate and report the minimum and maximum concentration at which the drug is sufficiently stable and to note any restrictions that this may pose for vascular access. For example, the need to use the central venous routes and slow rates of administration if higher concentrations might irritate or damage veins. The consequences of inadvertent extravascular administration should also be considered.

When drugs require dilution for administration the carrier fluid studied should include dextrose (5% and 10% *w*/*v*) as well as sodium chloride 0.9% w/v to maintain isotonicity. If dextrose solutions are suitable, this will help with sodium restriction and provide additional energy. Water for Injection should not usually be considered a suitable infusion fluid because of the potential risk of infusing hypotonic solutions, but information on stability and osmolarity may be useful if dextrose is inappropriate and sodium balance a problem.

Many neonatal patients in a critical care setting receive between 15 and 20 IV medications daily, the majority of these are unlicensed or used off label [4]. Lack of knowledge around the physicochemical incompatibilities of IV drugs in NICU and PICU settings often necessitates the use of a dedicated IV catheter in neonates and infants who have limited IV access [28,29]. Drug incompatibilities are often an underestimated aspect of clinical practice and are concerning in the neonatal population where a lower capability to compensate for adverse drug reactions may lead to higher morbidity and death [30,31]. This concern is exacerbated in neonates by the frequent requirements for polypharmacy, multiple infusions delivered through a single catheter due to limited vascular access, low infusion rates exposing drugs to longer interaction and the possibility of incomplete dissolution or precipitation of drug due to low volumes of drug solutions [32]. Realistically, limited venous access can result in little choice but to co-administer drugs.

A recent report highlighted the extent of the problem and reported that among medicines tested as a co-infusion of two drugs listed as a common NICU medication, only 4% of the combinations were fully compatible [28]. The compatibility of IV injections with other commonly administered drugs and infusion fluids should be studied when mixing at a ‘Y’-site when the drug is likely to be used in the intensive care situation.

#### 2.2.2. Oral Delivery

Medicine administration via the oral route to children less than two years of age can be difficult for both parents and children [13,33]. The main issue in administering oral medicines to neonates lies in their ability to effectively swallow the medicine. Typically, most oral processes are present from birth (rooting, lip, lateral tongue, mouth opening, biting, and emerging chewing behaviours). However, even oral syrups are not always fully swallowed when administered to neonates and infants [34]. This is further exacerbated in preterm neonates, where issues around swallowing and ADME (absorption, distribution, metabolism, and excretion) factors may be less well understood. Clinical evidence suggests, that the oral absorption process of a drug undergoes substantial changes after birth [35,36]. The impact of these physiological changes on oral drug therapy is currently unclear.

Historically oral liquid formulations have been used in neonates. Examples of commonly used oral medicines for neonates include vitamin D drops, analgesic suspensions (ibuprofen, paracetamol), antibiotics, glucose gel for treatment of hypoglycaemia and anti-reflux medicines. The first approved medicine to treat neonatal diabetes (Amglidia^®^) is a suspension formulation of glibenclamide [37].

EMA guidance [20] suggests, that the following oral formulation options are suitable from birth: Powders and granule (administered as a liquid) and oral liquid preparations (solution, suspension and oral drops).

It is important that appropriate strength medications are available for neonates to ensure that appropriate doses/volumes can be accurately measured and administered. The maximum recommended single dosing volume is 5 mL for children aged below five years and 10 mL for children aged below 10 years [38]. For neonates, dose volumes as low as 0.1 mL may be required.

The importance of accurate dosing to the youngest children has previously been highlighted [39]. Dosing devices for this age group mainly involves the use of an oral syringe or dropper though both have significant issues in terms of accuracy [40,41]. Therapeutic nipple shield [42,43] and medicine dispensing pacifiers [44,45] have been suggested for home use, but these are as yet experimental. In hospitalised patients, enteral tubes can be used for the administration of oral medicines where liquids (ideally solutions rather than suspensions) are the preferred dosage forms. Emulsion formulations can also theoretically be delivered via this route. One example is enteral nutrition or milk. There is a risk that the PK of a highly lipophilic drug, delivered via this route, may be altered if co-administered with an emulsion formulation or milk. Although during development compatibility of drug-enteral feed will be evaluated for new medicines, it is impossible to consider every feed available to review the likelihood of an interaction. Thus, there are circumstances where a drug–feed interaction may lead to PK variability as a result of a novel feed. Many medicines currently used in neonates are unlicensed for use in this population, and there is little or no information on their compatibility with milk or enteral feeds which may lead to a change in the anticipated PK. This is addressed in more detail in section (biopharmaceutical consideration).

Palatability considerations for neonates are more commonly associated with volume and texture of medicines in addition to taste [46,47]. However, some taste preferences seem to be innate (e.g., sweetness), and in extreme cases, bitter taste can lead to vomiting, so taste cannot be ignored even in neonates. New products that are designed for children are likely to be those that offer flexible dosing to target not only neonates but also older infants and children. Therefore, a product that meets the needs of all age groups will be developed which will address issues of palatability for infants and children.

#### 2.2.3. Rectal Delivery

Rectal administration is more commonly used from infancy onwards [48]. This route suffers in neonates from unpredictable absorption which seems to be the limiting factor [36]. This is highly correlated with faecal incontinence/retention of the drug dose, which is inversely related to age/maturation. Solid dosage forms are usually better retained in the rectum than liquids. However, flexible and accurate dosing, which is an important product feature, cannot be delivered when suppositories are split at the point of administration [49]. This does not completely prevent the use of the rectal route to reduce the need for parenteral administration, or overcome oral administration restrictions (e.g., physiologically reduced gastrointestinal absorption, nausea, vomiting, seizures, nil by mouth), but safety and efficacy with appropriate bioavailability studies and patient size-appropriate product are paramount. In fact, recent studies (pain, patent ductus arteriosus closure) [50,51,52] demonstrated similar effectiveness for rectal delivery (mainly small enemas) as compared with IV or oral delivery even in very low birth weight preterm infants as well as being cheap and safe. Therefore, in resource-limited settings, rectally formulated drugs for pre-referral use could have great potential, e.g., neonatal septicaemia, pneumonia or malaria [53]. Recently, rectal antibiotic (Ceftriaxone) to reduce treatment delays in neonatal sepsis presented as rectodispersible capsules have been proposed [54].

#### 2.2.4. Pulmonary Delivery

The respiratory route is not much used therapeutically in preterm and term neonates with one major exception.

Respiratory distress syndrome (RDS) is a life-threatening condition, which occurs almost exclusively in preterm neonates with a deficiency [55,56], dysfunction or inactivation of pulmonary surfactant. The physiological role of surfactant is to allow the lungs to expand and avoid collapse (atelectasis) during the expiratory phases. Lack of surfactant results in difficulty in breathing, with low oxygenation, increased breathing effort and the need for respiratory support.

The administration of exogenous surfactant can alleviate the symptoms of RDS by supplementing the endogenous pool of surfactant, thereby enabling the biofilm to be replenished, dramatically reducing mortality and morbidity. This is often administered via invasive methods, such as endotracheal intubation and mechanical ventilation (MV). The gold standard version of this approach [57,58] is called INSURE (intubation, surfactant administration, extubation). Less invasive approaches, using thinner catheters (such as LISA technique—less invasive surfactant administration [59,60]), have been designed to supply exogenous surfactant to spontaneously breathing neonates, but these are still partially invasive. Therefore, there is a great interest by neonatologists in the development of a truly non-invasive procedure for surfactant administration, such as nebulisation. Some interesting pilot studies have been published on this issue [61,62,63].

These various intubation techniques also allow administration of other therapeutic interventions to treat a wide range of breathing difficulties should these be required. It is important that any formulations administered in this way, are of the correct concentration to be able to deliver the required dose in a small volume, and that the rheological profile is adequate to permit flow through the narrow tubes involved.

For neonates who can breathe spontaneously the major route of drug delivery is via nebulisation. A wide range of breathing difficulties can be treated, including respiratory syntical virus (RSV) bronchiolitis or asthma-like reactive airways disease. Typical drugs administered include beta agonists, steroids, ribavirin and sometimes adrenalin.

An advantage of using the pulmonary route is that efficacy can be achieved with reduced systemic drug levels and hence, decreased side effects [64]. For example, a recent review [63] has shown that pulmonary administration of corticosteroids can effectively prevent bronchopulmonary dysplasia in preterm infants with RSD, without the adverse effects on growth and neurodevelopmental outcome associated with systemic delivery. An emerging practice consists of adding budenoside to surfactant [65].

The formulation for nebulisation is usually a sterile aqueous solution or suspension, mainly based on the solubility and stability proprieties of the active drug substance. Many times, in order to achieve the suitable physico-chemical characteristics (i.e., osmolality, viscosity, pH etc.) and stability, it may be necessary to include excipients in the formulation. However, only a few excipients are approved for the inhalation route, and usually, there is little if any specific safety data available for neonates or the wider paediatric population [15], let alone after pulmonary exposure. Nebuliser solutions should be presented in an age-related unit dose of appropriate concentration if possible. Where this is not possible, the device used to measure the dose must not be a syringe designed for injection, thus reducing the risk of the nebuliser solution being inadvertently injected. The most appropriate nebuliser system to achieve good inhalation performance in terms of output rate, dose delivery to the lung, aerodynamic particle/droplet size distribution (i.e., fine particle or respirable fraction below 5µm or 3.3µm) and respirable drug delivery rate must be chosen. It is worth highlighting that the lung deposition of a nebulised product can be influenced by the breathing pattern (i.e., tidal volume, breath frequency and inhalation/exhalation ratio). The breathing pattern depends on the patient’s age, and so, neonates (preterm and term) and children have significantly different and variable breathing profiles, making reliable dosing difficult when the efficiency of drug delivery systems are breathing pattern dependant [66,67]. This is further complicated if the infant is crying as this also changes breathing patterns.

It is possible to model the influence of various anatomical, physical, and physiological factors on aerosol delivery in preterm neonates on the efficiency of the delivery of an aerosolized drug to the bronchial tree using 3D models such as the (PrINT model) [68].

Nebulised delivery may also be important in older paediatric patients during times when the child’s inspiratory force is low such as during an acute exacerbation of their condition. However, for both babies and older paediatric patients, some studies have shown that drug delivery from a Pressurised Metered Dose Inhaler (pMDI) and spacer system with suitably sized facemask can be at least as effective as nebulisation [69,70]. Delivery via pMDI and a spacer system is recommended in NICE guidance [71] for routine treatment of older paediatric patients.

#### 2.2.5. Nasal Delivery

Although the nasal aperture is small in diameter in neonates and the nasal mucosa is often delicate and coated with mucus, it nevertheless offers a potential route for employing local drug administration to effect systemic drug delivery, thus reducing the need for injectable administration. In emergency care, this could also decrease the need for additional painful procedures such as insertion of IV cannulas for medication administration. For example, intranasal fentanyl has been used in the palliative care of term neonates and infants [72]. As such, it has attracted significant research interest in the last few years for use in this age group. Even if intubation is required, nasal administration could provide a less impactful means of aiding that procedure. Nasal midazolam was used and was more efficient than nasal ketamine, to adequately sedate neonates requiring intubation in the delivery room [73]. A typical dose volume was 0.1 mL/kg in each nostril.

Potential advantages of this route of administration, are that it is less invasive than IV whilst maintaining rapid onset since medications are directly absorbed (parenterally) through the nasal mucosa into systemic circulation, it also results in higher bioavailability compared to oral medications, as first-pass hepatic metabolism is bypassed. There is also some evidence that brain penetration can be enhanced as a consequence of nasal delivery [74,75,76].

Compared to buccal administration, (another method of avoiding first-pass metabolism), where salivation and swallowing issues after administration in neonates could have a substantial impact on dosage accuracy, more consistent absorption and bioavailability have been obtained following intranasal administration [77].

Bypassing the gastrointestinal tract with nasal drug delivery allows for the delivery of some macromolecules with low permeability across the gastrointestinal tract or susceptibility to chemical/proteolytic degradation. Recent studies in neonatal mice highlighted the potential of intranasal immunization of neonates with live vaccines [78]. As vaccination at birth would provide early protection for neonates and infants, expanding and improving the available means of neonatal vaccination is a global health priority.

There is a well-established link between nasal dosing and enhanced blood–brain barrier (BBB) permeability. This may or may not be of therapeutic benefit. If central activity is not desired, then nasal dosing may lead to enhanced toxicity. The BBB is a dynamic physiological barrier which regulates the passage of hydrophilic molecules into the central nervous system (CNS) via a physical barrier formed by tight junctions (TJ) between the endothelial cells and also a system of influx and efflux transporters and enzymes. Recent reviews and papers on the development of the BBB in preterm and term neonates suggest that although the physical barrier is formed very early, many other factors that affect brain penetration favour increased drug levels in the brain and cerebrospinal fluid (CSF) and that these factors change rapidly with maturation [79,80,81]. As yet, there is little or no information on the effect of actives and excipients on the development of the BBB in these age groups. It is known, however, that some penetration enhancers used in intranasal formulations for adults work via breaking tight junctions which is clearly not safe in neonates [82]. Conversely, cyclodextrins (e.g., hydroxypropyl-β-cyclodextrin), which are increasingly being used as vehicles to transport lipophilic drug through BBB [83], may have a neuroprotective effect and are used therapeutically in the treatment of neonatal hypoxia-ischemia [84].

Alterations in BBB development and in TJ expression could lead to anomalies later in life as well as to increased predisposition to some diseases. Neonatal hypoxia-ischemia (HI) causes severe brain damage and remains a major cause of neonatal morbidity and mortality [85]. At present, treatment options for neonatal HI brain damage are very limited and have only modest efficacy [86]. A study conducted in several rodent models of ischemic brain injury demonstrated the therapeutic potential of mesenchymal stem cells (MSCs) transplantation that improves functional outcome and also restores brain structure [87]. These findings in rodents indicate that the nasal route was an efficient route for stem cell transplantation after brain injury in the neonates [88].

During the development of a new formulation for nasal administration to the neonate, as well as all the usual factors (e.g., pH, osmolality, chemical irritation and overall acceptability) the formulator needs to keep in mind:

- The ratio between the ideal volume per nostril and the concentration of solution/ suspension to be administered. In practice, the maximum volume for single administration into one nostril is 0.1 mL in neonates and 0.5 mL in older children [89]. There is no agreement about the volume that can be given to preterm neonates. Larger doses can be given by using these dose volumes (or half the total volume provided this does not exceed the safe total volume) in both nostrils.
- The need for a ‘baby size’ device able to dose accurately very low volumes of liquids without causing physical damage to the nasal mucosa.
- The potential irritancy of highly concentrated solutions, especially if these are hypertonic.
- The choice of excipients. For example, a penetration enhancer may be required to aid the absorption of polar drugs. Many of these could cause irritation of nasal epithelium of neonates, and for most common penetration enhancers, no safety data are available in the neonatal population.

#### 2.2.6. Dermal and Transdermal Delivery

In neonatal practice, many of the topical treatment decisions are made by specialist nurses. It is therefore very important for formulators to provide a high level of support to enable safe and effective use of topical formulations.

Formulators should be aware that the skin barrier may not be fully formed in preterm infants both at birth and for up to four weeks afterwards [90]. There are also changes in the type and proportion of lipids present and the development of ancillary skin structures such as sweat ducts and hair follicles [91]. This has implications for potential toxicity of active pharmaceutical ingredients (API’s) and formulations usually delivered for a localised effect via the topical route. In the absence of a fully competent skin barrier, higher systemic exposure of both API and common topical formulation excipients could be expected. The skin barrier may also be breached in term neonates and infants due to conditions such as cradle cap, nappy rash and eczema/dermatitis. Even if the skin is fully formed and functional, the lipid composition changes rapidly and the stratum corneum tends to be thinner in young babies and more hydrated. Additionally, occlusive dressings including nappies/diapers with impermeable plastic coverings can increase absorption. Besides permeability, the simple BSA/kg ratio puts neonates at a higher risk of increased absorption. Therefore, any adverse effects tend to be exacerbated by the relatively higher surface to volume ratio in children leading to a risk of unwanted high systemic exposure if large areas of skin are treated.

Formulators, therefore, need to ensure that APIs delivered topically in neonates have excellent systemic safety (limitation to the skin alone cannot be assumed). It is insufficient to only undertake topical safety studies as systemic absorption should be assumed and therefore systemic safety studies are required. Toxicity of ingredients in subpopulations such as neonates requires a careful risk assessment to avoid the use of excipients with unclear safety in target population. It is also necessary to select formulation excipients that are intrinsically safe and have a low potential to cause irritation or other skin reactions, e.g., sodium lauryl sulfate [92].

Finally, it is important to ensure that the pH and tonicity of the formulation is well matched to the specific requirements of the skin at the various stages of development and an understanding that this can change rapidly [93,94].

Coupled with the state of development of the barrier function of the skin, it is important to remember that skin in the neonate is often fragile and that it can be damaged by mechanical abrasion. Thus, the rheological profile and other cosmetic attributes of the formulation need to be taken into account. It may well be desirable to use a specific neonatal formulation that is more fluid/easier to spread than is common for creams and ointments used in older children. Similar considerations apply to the potential skin damage that can be caused by adhesives used in transdermal patches.

In some cases, the topical route is deliberately used to achieve systemic delivery [91]. In this case, it is still important to remember that the barrier properties of the skin may be rapidly changing during early development as this might affect the systemic blood levels achieved from a particular dose with implications for the topical dose that needs to be given.

Indeed, dose flexibility is a challenge in the neonate due to the above-mentioned developmental factors specific to the skin and due to the rapidly changing weight of the neonate. For standard semi-solid formulations, this can be accommodated to some extent by the area that is treated, but a wide range of strengths is still likely to be required. This is often achieved by diluting a formulation extemporaneously with a base. If this is done the stability implications (chemical, physical and microbiological) need to be considered along with the issues of ensuring homogeneity of the diluted formulation. For ‘unit dose’ topical formulations such as patches, dose control is achieved either by cutting patches or masking them (usually off-label) to reduce the contact area. Both practices are subject to significant risks of poor dose control unless the developer fully validates this practice.

Once a fully competent skin barrier is formed, it is possible to use the full range of topical and transdermal delivery [91] including creams, ointments, sprays, lotions, baths and, less frequently, transdermal patches with suitable adjustment to the dose. Some patches have paediatric labelling supported by clinical trials, whereas others are used off-label. There is little literature on the utility of innovative delivery methods such as needleless injectors, iontophoresis, sonophoresis and microneedle patches in the neonate. However, there is evidence of them being tested for use in older paediatric patients in order to attain more reliable drug levels, either locally in the skin or systemically, that are bioavailable from creams and ointments, especially where dermal permeation of the API is low or slow.

### 2.3. Ability to Administer: Product Factors to Consider

The lack of appropriate dosage forms frequently results in off-label and/or unlicensed use of modified adult formulations for administration in neonate and paediatric patients [95]. It has been reported that between 71% and 100% of patients in the NICU receive at least one off-label or unlicensed medicine [96].

The dose of a medicine can vary 100-fold between that for a preterm baby and an adult. Whilst a liquid medicine may be acceptable for all ages, using just one concentration for all ages would mean that an appropriate dose volume for an adult (say 10 mL) would be impossible to measure accurately for a preterm neonate (say 0.1 mL). Thus, more than one concentration would be required in this example.

Fortunately, many medicines can be dosed according to weight bands (e.g., 5–10 kg, 11–20 kg) or by age bands making it easier to produce an acceptable standard concentration of a drug. Formulations available from manufacturers should follow this rule, making it more feasible to manipulate with drug doses for neonatal application. However, doses of potent medicines for neonates necessitates individualised dose calculations based on body weight (e.g., mg/kg) or body surface area (e.g., mg/m^2^). This could lead to the preparation of very low drug concentrations, several manipulations in preparation of the end formulation and the management of multi-infusion IV administration [97]. This has been reported to lead to a significant risk of medication errors and adverse drug events, especially in the NICU [98].

To overcome these issues associated with individualised concentration calculations based on low body weight, formulations of standard concentrations [97,99], specifically of high alert medication, have been implemented into NICU settings [100,101,102]. These can then be used with intelligent infusion pumps programmed with the standard concentrations allowing automated calculation of the individual flow rate based on body weight. Standardised concentrations also make it easier for pharmacies to prepare or source drugs which are ‘ready to infuse’ rather than them being prepared at the bedside with the attendant risks. They also reduce the burden of complex patient-individualised calculations on the prescriber and nurse administering the medication [97,99,103] and standard concentrations of different strengths allow for flexibility to compensate for a patient’s differing fluid requirements, though the existence of more than one concentration introduces the potential for product selection error.

As the flow rates of standard concentrations are linked to patient weight, the flow rate decreases with decreasing patient’s weight. This can result in very low flow rates in extremely low birth weight neonates where rates may be as low as 0.02 mL/h. Low flow rates have been associated with long delays in drug delivery times and hence the onset of effect, delays in transitioning to new infusion rates and delays in reducing effects when the infusion is stopped [27]. Such unpredictable delivery is unacceptable in the administration of life-sustaining medicines to critically ill patients [104,105,106]. Lack of awareness of these issues among clinical staff may lead to inappropriate clinical decision-making [27,104]. Observed lack of response to treatment due to delivery delay may cause precocious dose increase leading to overdosing and toxicity. When examined together with low flow rates, several other factors may contribute to prolonged and unpredictable delivery such as, the dead space of the administration system [27,104], the adsorption of the drug to tubing, backflow of the infusion [105], change of the flow rate of other infusions or a carrier fluid [27], the type of pump used, the placement of the infusion tubing and the line architecture [27].

When designing new formulations or reformulations of existing drugs, clinicians and drug formulators should work together to maximise an optimal drug delivery under special neonatal drug administration conditions (Table 2). Information and advice provided to the end user should facilitate a practical dosing regimen taking into account the potency of the drug and potential adverse effects and the setting in which the drug is to be measured and administered (NICU, hospital ward, home).

Using standard concentrations of medicines can help avoid medication errors when patients move between care settings [107]. Due to the implementation of standard concentrations to the neonatal care, the number of individually prepared drug doses reduced dramatically. Formulation of medication strengths that could represent standard concentration used in the NICU would eliminate the step of drug manipulation to prepare standard concentrations.

#### 2.3.1. In use Stability Issues

As well as all the administration factors discussed above, it is clear that formulations need to be stable in order to deliver the expected dose to the patient.

Although all the usual ICH stability requirements will apply, the therapeutic environment will often present some additional ‘in use’ stability challenges that should be considered by the formulator when developing products for this age group. This is particularly true for preterm infants and those being treated in high dependency incubators or intensive care environments and when those infants are treated almost exclusively via the parenteral route. They are complex to anticipate and therefore to reproduce in vitro despite regulatory expectations. Yet, conditions in NICU may be more controlled than in other environments. Clinical colleagues can provide information on likely treatment scenarios that will allow the most common potential interactions to be mimicked. Drug concentrations, diluents, formulations, mixing ratios and environmental conditions (light and temperature) are some factors pertinent to the design of compatibility studies. Some of the major issues are true pharmacy issues that need to be considered within the clinical context:

a) Photostability—useful reviews have been published [111,112]. Light intensity is often higher in the neonatal environment. Phototherapy is often used to treat postnatal jaundice either with lamps having the required spectral power distribution to break down the bilirubin in the skin or even exposure to direct sunlight. The implications of this treatment to the stability of any drug that partitions to the skin or eye need to be considered along with the implications for photosafety particularly if the metabolic immaturity of the neonate might be expected to affect clearance of any photoproducts that are generated. When bilirubin is present in the skin, protein binding will also be high. This can either exacerbate or mitigate photostability issues, depending on the mechanism of photodegradation. It is extremely difficult to predict what effect this protein binding might have *a priori.*

If the infant is being treated parenterally, it should be remembered that solution concentration tends to be low (though they can also be high if the neonate is fluid restricted) and infusion rates can also be low leading to long residence times in the administration lines. There is often a transit time of several hours between the infusion pump and the patient [27,106]. Added to this is the potentially very large surface to volume ratio due to the solution flowing through a long narrow bore tube. All of these factors can lead to significant levels of photodegradation that may not be fully predicted by some ICH Q1B photostability tests. It is therefore important to undertake a well-designed in use photostability test to allow for suitable advice to be provided to the healthcare practitioner.

Another factor that needs to be considered here is that the material of construction of any transfer line may be quite variable. Thus, some plastics will contain quite high levels of UV stabilisers whilst others may have much lower levels or even none at all. Clearly, this can lead to different levels of photoprotection for the content of the line.

If the product is known to be photolabile, then it may be wise to advise that any holding vessel (such as a syringe or infusion bag) and particularly any transfer lines are shielded from light exposure either with a suitable coloured cover or with a light impermeable barrier such as aluminium foil. One downside of doing this is that it will render visual inspection more difficult.

b) Other environmental factors—due to potential difficulties in breathing and less developed thermoregulation preterm infants may be in an oxygen-rich environment that is maintained at a higher temperature than would usually be the case in the hospital environment. This has obvious potential implications for formulations that are subject to oxidation or thermally driven hydrolysis if they are stored in such an environment for extended periods. Again, this may be further exacerbated by low concentration. The formulator will need to consider whether or not these conditions might have an effect on their product and provide suitable handling advice accordingly.

c) Potential Interactions—due to the limited number of access points for parenteral delivery to the neonate, it is often necessary to provide several drugs via the same entry point. In addition, neonates may be provided with nutrition via the same route. Thus, it is important to consider potential incompatibilities (both chemical and physical) between the various medications and the potential for API to adsorb to components of the parenteral or enteral feeding formulation used. In some cases, high concentrations of dextrose may be administered in this age group, both of which could have effects on product stability. On top of that, there is a risk that the API may interact with the material of the transfer tube leading to adsorption of the API. If the concentration is low, there may be a significant loss of therapeutic activity.

d) It may be a challenge to provide a sufficient range of formulation strengths to cover all needs of the paediatric population. Dilution may be required to allow accurate measurement using routinely available syringes. Large dilution factors may be required if only a single product concentration (often designed for adults or older children) is available. If dilution is to be avoided it may be necessary to develop and provide an appropriate dose measurement device and/or advice on providing an accurate dosage. Another method for controlling dose (volume) delivery is to change the infusion rate as discussed in the parenteral section.

If the formulation contains a stabiliser of some sort (e.g., antioxidant, photostabiliser) then dilution may render that stabiliser ineffective with obvious potential consequences for the formulation stability. The extent of the impact will be determined by a knowledge of the stability of the unprotected API and the level of stabiliser that remains. If the parenteral product is an emulsion or suspension, then dilution may lead to physical instability potentially leading to blockage of cannulas or even phlebitis. On the positive side, dilution may also reduce intake of an excipient that might be harmful to the neonate, e.g., sodium metabisulfite in parenteral inotropes.

The diluent used is also of importance. In some instances, photodegradation of the diluent can lead to degradation of an otherwise photo stable API (e.g., [113]). Formulators should be aware of this possibility when considering which diluent to recommend.

Photostability (and possibly oxidative stability) is also of concern for formulations that are applied topically in such a high-intensity light environment. Clearly, there is a risk that stability could be poor, leading to suboptimal dosing and/or the generation of relatively high levels of photo products in situ. The fact that the API concentration could be low should be considered when assessing the likely impact.

#### 2.3.2. Excipients

Some of the challenges in the development of formulations for neonates have recently been reviewed by Kogermann et al. (2017) [114]. Not surprisingly, this review focuses largely on pharmaceutical excipients since mainly liquid dosage forms are used in these vulnerable patients and these may require preservatives to limit microbial growth and/or an array of excipients to achieve a solution of the active or to formulate a palatable liquid. Given that most actives are not specifically developed for neonatal conditions, dosage forms may contain excipients deemed safe in adults or older children that have not been studied for neonates [14,15].

The immaturity of organs and physiological systems of the neonate is important for the ‘handling’ of excipients as well as active drug substances. Little is known or published about the acute or chronic effects of excipients in the very young and that which has been published indicates their vulnerability to toxic effects considered safe for older children and adults. The exposure limits for many excipients have been published, but often apply to adults and should not be applied to neonates unless specifically indicated. Non-clinical work in appropriate juvenile animal models may be required for excipients used for the first time in the very young, and there should be appropriate justification for the use of any excipient supported by studies of high quality.

Epidemiological studies have demonstrated the exposure of neonates to excipients and the variation between products marketed in different countries [115]. Studies have also shown that excipient exposure can exceed current safety limits and that exposure may be from several medicines [116]. Measuring blood levels and the kinetics of excipients is possible in the very young and can be contemplated as part of clinical studies if necessary [117].

The formulator should first consider whether any excipient is required when developing a formulation for neonates. For example, it should not be necessary to include an antimicrobial preservative in a liquid product if designed for single use. Even an oral liquid medicine might be presented as a sterilised unit dose preparation without preservative excipients. Colours are generally not needed for this population and if intended for administration via enteral tubes, sweetening and taste masking may not be required. A risk-based approach [118] taking into account PK, ADME and safety data relevant to neonates may be taken in discussion of potential formulations with clinical and toxicology colleagues. For example, an excipient toxicity profile acceptable for single dose administration for a life-threatening illness may be quite different from that for long-term administration for a condition for which effective remedies are already available. Of course, the overall cost of formulation development for neonates and the viability of the commercial product will also require consideration during this pre-formulation discussion.

The European Paediatric Formulation Initiative (EuPFI) STEP database [119] provides comprehensive information on excipients for children and is freely available. The European Commission, through the EMA, is revising the labelling requirements for excipients in authorised products. Supporting the thresholds for labelling requirements are extensive reviews of excipient properties, uses and toxicology [120]. Whilst not intended to provide direct guidance to the formulator, that for propylene glycol [121] serves as a good example of the way in which toxicity can be assessed and safe exposure limits calculated.

As with any product development, compatibility studies of any proposed excipients with the API are an important part of early product development.

#### 2.3.3. A Shift towards Solid Dosage Forms?

The mainstay of oral product administration for neonates are liquid dosage forms. Despite being more common for the paediatric market, they tend to contain excipients with elevated toxicological risks in neonates compared to adults (e.g., ethanol, propylene glycol, benzyl alcohol, polysorbate, parabens, etc.) due to the ongoing organ development and incomplete maturation [122]. REF oral solid dosage form is by far dominating the adult market [123]. Although they may sometimes contain excipients with some toxicological concerns of their own (for example, some may be allergens or irritants), in general, excipients used in solid dosage forms have a more benign safety profile than some used for liquid medicines [124]. As discussed above (Section 2.2) neonates are not always able to swallow liquids let alone solid dosage forms even though there are many inappropriate products on the market such as capsules and tablets licensed from birth that will have to be manipulated for administration [125]. It is highly unlikely that this is based on clinical data and hence, would no longer be acceptable following the implementation of new regulations worldwide.

The attraction of solid dosage forms has led to the emergence since 2008 of flexible solid oral dosage form (FSOD) which has been actively promoted by the World Health Organization (WHO) to overcome challenges of ensuring access to suitable medicines for children [126].

FSOD are solid forms that do not have to be swallowed whole, such as dispersible tablets, effervescent tablets, orodispersible tablets, and sprinkle capsules. They are flexible in administration, though not necessarily in dose. They may provide a convenient dose unit that can be manipulated to provide a suitable form for delivery to a neonate.

For example, some tablets for older children can be cut accurately into two, four or even eight approximately equal pieces. Such tablets were developed for fixed-dose combination of zidovudine and lamivudine in fast-disintegrating subunits (per 5 kg of BW) [127]. These can be easily administered after dispersion in a liquid or milk.

Dispersible tablets offer more dose flexibility. However, the minimum adequate volume of dispersion linked to the reproducibly of accurate dose withdrawal needs to be considered concomitantly [128]. In theory, such a dispersion could be dosed via an enteral tube. The suspension should also of sufficiently low viscosity and be fine enough to pass through the enteral tubes if this is foreseen.

As effervescent tablets have high-sodium content, their use should be carefully considered for neonates. They are also clearly contraindicated in cases where the patient in hypernatraemic, who need to be sodium restricted. Dispersions probably need to be degassed prior administration.

Some tablets have been developed to be administered in a Therapeutic Nipple Shield to safely deliverer medications and nutrients to breastfeeding infants [129]. In one proof of concept study, formula or freeze-dried milk/drug tablets were formulated to demonstrate both reliable drug delivery to babies and the ability of milk to dissolve poorly water-soluble drugs [130,131]. Both are interesting concepts considering babies’ exclusive milk-based diet. There is also some evidence that milk can mask the taste of some medicines. However, the risk of interfering with feeding by rendering the taste of the milk foul needs to be mitigated with decent palatability of the medicine itself.

**Figure 2:**
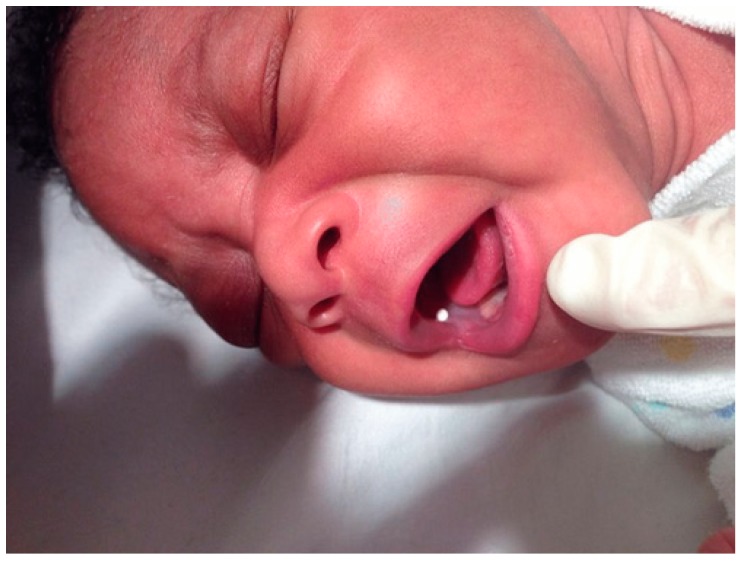
Newborn child with uncoated mini-tablet in the cheek pouch before swallowing (with permission to use from Thabet et al. 2018 [132]).

Until recently, few studies have been undertaken on the acceptability of mini tablets for patients under six years of age. Recently, however, a study in 6 to 12 month-old children that also included some neonates (*n* = 151) demonstrated complete swallowing of one mini-tablet (82.2%) compared to 72.2% for a liquid syrup [132] (Figure 2). However, it is to be noted that this mini tablet was uncoated so would have dispersed if it were inhaled inadvertently rather than swallowed. This was for safety reasons but may have somewhat confounded the study. On the positive side, there was no evidence of choking. As this has been a concern for some time, it would appear that the acceptance of this dosage form has come a long way. Data on acceptability and swallowability of several hundred mini tablets in slightly older babies, infants and children (six months to six years) are awaited. In parallel orodispersible minitablets that can be dispersed in the mouth or in baby-friendly beverages to particles that are easy to swallow are proposed to reduce the risk of choking while allowing dosing via nasogastric tubes (NGTs) [133].

Other orodispersible dosage forms, such as thin polymeric film, have been studied in older age groups (0.5–6 years old) [134]. They are attractive as they overcome the need for swallowing a solid entity. However, there is no precedent of use in neonates. This is also true of micropellet formulations such as sprinkles. It is hoped that work with older babies will provide usability and safety evidence and translate in product development in the near future that could benefit neonates too.

### 2.4. Ability to Administer: Device Factors to Consider

#### 2.4.1. Accuracy of Small Volumes

Measuring small volumes (e.g., bolus IV injections, oral liquids, low rate IV infusions) with sufficient accuracy can be problematic if routinely available administration devices such as oral/enteral syringes and injection syringes are to be used [40]. Using a liquid product with a concentration designed to administer standard volumes to adults or older children may mean that volumes of 0.1 mL or less might be required for neonates [39,40,135,136,137,138,139]. This issue can be even more acute if clinical practice standardises on a single oral/enteral syringe design rather than using any specific device that may be provided by the manufacturer. To mitigate this issue, formulators should ensure that they develop the right product strength(s) so that there is no need to measure small volumes, especially that no volume is less than 0.1 mL and at the same time, the strength allows to cover body weights from 0.5 kg to 5 kg (a 10-fold range).

Calculating the required dose volume may be difficult, and dilution steps may add to calculation errors and be undertaken in an unsuitable environment. Decimal fractions involving hundredths of a mL can be confusing.

For injections the volume presented in a container should not be greater than ten times the dose for the smallest child and for drugs given by other routes of administration the risks of miscalculation or inaccurate measurement should be risk assessed and steps taken to reduce the risk [20].

It would be helpful if the device industry could develop administration devices that can accurately measure and reproducibly deliver very small volumes.

#### 2.4.2. Enteral Tubes Administration

Neonates may require enteral feeding tubes to allow safe administration of enteral feeds, fluids and medicines. Older children with swallowing difficulties (sometimes related to administration of medicines only [140]) may have enteral feeding tubes inserted at different sites in the upper gastrointestinal tract for the long-term administration of food, fluids and medicines. The likelihood of administration via an enteral tube is one of the factors that will determine the investigations required to demonstrate that the drug preparation can be delivered effectively via the tube and without adverse effects. The flush volume required to ensure the whole dose is delivered must be carefully considered in relation to fluid requirements. If the product is likely to be administered via an enteral (e.g., nasogastric, nasojejunal) tube, issues such as viscosity of formulation (to permit flow of the product through neonatal tubes [e.g., 6FR/8FR] and avoid blockage), size of particles, adsorption to commonly used enteral tubes and interaction with common formula/breast milk should be investigated [20]. A recent Q&A has been produced by the Quality Working Party of EMA and sets out the potential problems of administration of medicines through an enteral tube and the steps to be taken to investigate and minimise them [141].

#### 2.4.3. Parenteral Catheters and Administration Sets

An important formulation development consideration is to assess the risk of interactions between certain drugs and the materials of the devices used to administer them. Neonatal catheters and cannulas can be made of a variety of different materials including silicone, polyurethane, polyvinyl chloride and polyethylene [142]. Other components of the administration set may well be constructed from a different polymer to that of the catheter being used. Thus, the nature of the incompatibility may be complex. For this reason, the formulator should be aware of the composition of medical devices and potential incompatibilities.

The most commonly observed interactions are chemisorption, mechanical trapping of drug molecules onto or within the device and leaching. The occurrence of an interaction depends on the chemical nature of a drug and of the device material [143], the number of binding sites on the material’s surface (surface area, device dimensions) [143], the drug concentration [144], and the time of contact between the drug and the material’s surface which is influenced by the flow rate and the length and diameter of any tubing. The pH of the formulation may influence binding by changing the ionisation state of the API and any binding sites on the material of construction of the administration device. It is also important to consider any physical entrapment due to sharp bends in the tubing or via in-line filters. In the latter case, the pore size and material of construction could be important. Where medications are used at low neonatal concentrations, the loss due to interactions may be relatively significant [104]. A well-studied example is insulin [143,145,146,147]. Insulin adsorbs to glass and plastic polymers, binding more strongly at low flow rates to polyvinyl chloride (PVC) than to polyethylene (PE) tubing [143]. The opioid analgesic fentanyl and some benzodiazepines (diazepam, clonazepam) used in neonatal population for sedation and seizure control have been shown to interact with PVC IV lines and bags whilst there was no interaction with PE [144,147].

Another issue to consider here is that of the potential risk posed by extractables and leachables from any polymeric components. This issue is not restricted to parenteral delivery but may be expected to have a significant impact in this patient group. Leachables may be an issue not only for administration sets but also for primary, secondary and tertiary packaging. Table 3 below shows the likelihood of packaging component interactions.

Term (and to an even greater extent preterm) neonates are at higher risk of increased sensitivity to toxicants due to their organ immaturity and altered metabolic function [149]. This should be taken into account when establishing the safety threshold of any extractables and leachables. Official guidance on how to do this is available [150]. This includes the calculation of ‘safety factors’ for neonates and children but does not cover preterm infants.

With the advent of ever more sensitive analytical techniques, the number of compounds that may need to be evaluated, albeit at trace levels, makes this aspect of product development very challenging. It can, however, have significant effects in clinical practice.

As an example, some drugs, such as amiodarone and etoposide and some formulation excipients, such as those used in lipid emulsions, are known as leaching promoters [104,151]. Diethylhexyl phthalate (DEHP) is a plasticiser that is commonly used in PVC polymers that may be used in administration sets [152]. Animal studies have shown that high levels of phthalates may cause a range of issues in animals, including cancers, endocrine disruption and kidney injury [153]. The US Department of Health and Human Services issued a report in 2006 in which the risks of DEHP to human reproductive and development was evaluated [154]. In the report, neonatal patients and in particular pre-term neonates were highlighted by the FDA and the European Commission as being particularly at risk of exposure to levels of DEHP where there may be toxicological concerns [155]. However, DEHP free administration sets may not always be readily available. Lala et al. have described a case where an urgent need for an IV amiodarone infusion (which was infrequently used by their unit) was delayed several hours whilst attempts were made to source DEHP free components to adapt administration apparatus to avoid leaching of DEHP [104].

#### 2.4.4. IV Polypharmacy

**Figure 3:**
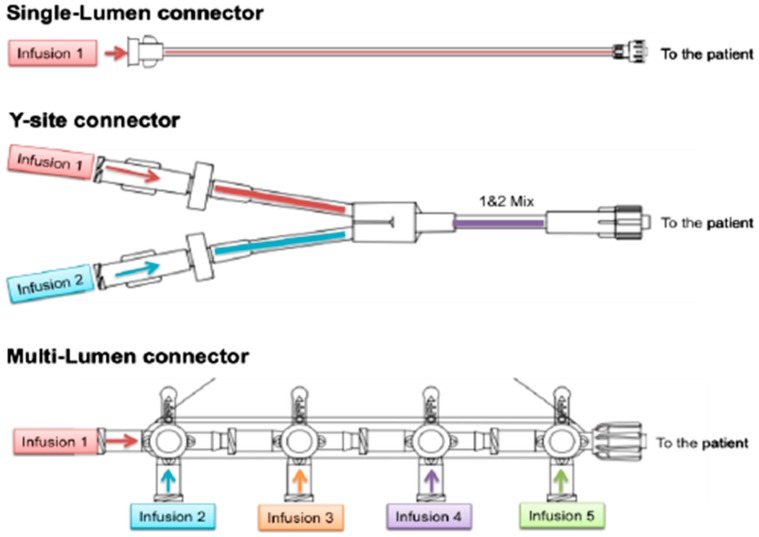
Different types of connectors (adapted from IV Sets and Access Devices Product Catalog—B. Braun Medical Inc., effective August 2017).

Patients cared for in intensive care units frequently require multiple IV infusions and present with limited vascular access. To avoid mixing different drug solutions in one IV container and to reduce the contact time between these drugs Y-site, T- or multi-lumen connectors are used to separate drug administration [31,156,157]. Some examples are shown in Figure 3 below.

**Figure 4:**
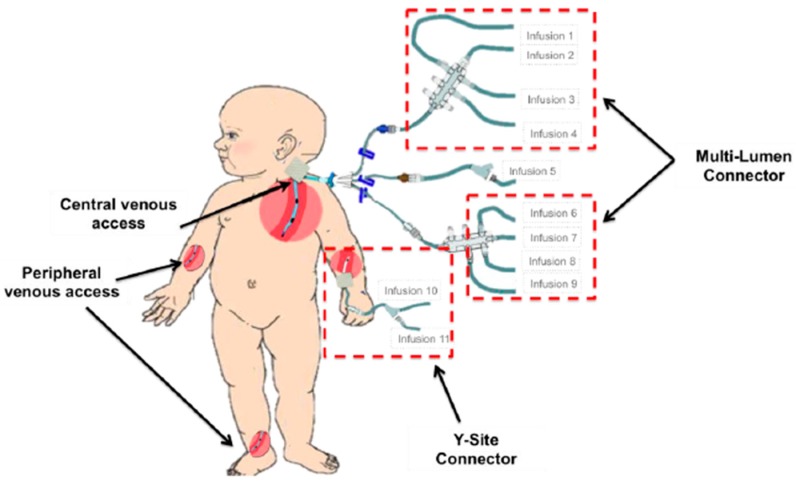
Example of venous access and multiple drug administration devices.

In practice, multi-lumen and y-site connectors are often used to simultaneously deliver multiple compatible drugs through one port of entry. The use of these devices can allow for multiple drug infusions to converge and be administered through a single venous port, if compatibility studies are available. This is illustrated in Figure 4. However, the optimum geometry, effect of mixing, flow resistance and risk of inadvertent boluses due to drug flow through these parts have not yet been sufficiently studied and compared.

#### 2.4.5. Inhalation Devices

**Figure 5:**
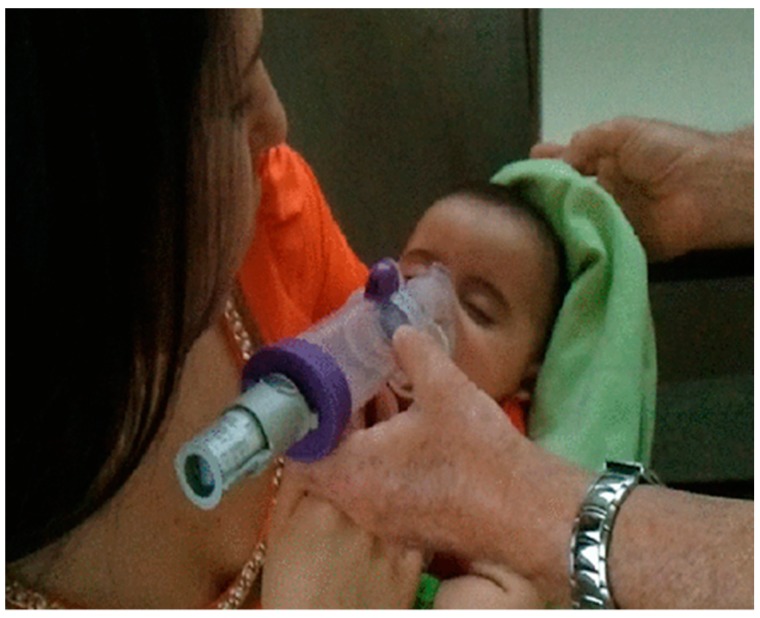
Photograph illustrating the method of aerosol administration to a sleeping infant showing the Respimat inhaler, InspiraChamber and SootherMask. (reproduced from [160] with permission from BMJ Publishing Group Ltd.).

The device used is an integral part of dose delivery of respiratory medicines, and hence, devices have been discussed to a significant extent under the respiratory product development section. Metered dose inhalers (MDI) need to be assessed for the safety of any extractables and leachables, e.g., from polymeric components of valves used in MDI. Aqueous formulations present less of a risk. Nevertheless, there have been some notable innovations in the use of devices to aid drug delivery in unusual settings such as the delivery of aerosolised therapy to a sleeping infant (Figure 5) and around device development [158,159].

### 2.5. Biopharmaceutical Considerations

The rate and extent of absorption, distribution, metabolism and excretion of drugs in children are different from that encountered in adults with the greatest differences from the adult being observed in neonates [161]. The impact of a number of biopharmaceutics factors on product development has been discussed inter alia during the discussion about the development of products for the various routes of administration.

Rectal absorption in full-term neonates has been reported as excellent. Intrarectal pH was shown to be significantly lower in neonates (6.5) compared with infants (6.9), and illness had no effect [162]. Neonates’ erratic absorption seems to be the limiting factor for wider use [36].

When medicines are administered via inhalation, breathing patterns can have an effect on the bioavailability and crying (which is more prevalent in neonates compared to adults) can reduce the amount of dose absorbed.

The skin barrier may not be fully formed in preterm infants, there are changes in the type and proportion of lipids present, and the development of ancillary skin structures such as sweat ducts and hair follicles differs to that of the skin of a term neonate. Skin may also be thin, fragile, hydrated and prone to occlusion. This has implications for potential toxicity of APIs and formulations usually delivered for a localised effect via the topical route.

Altered or reduced distribution is likely via most parenteral routes.

A recent review describes these differences in detail [13], the most relevant information has been extracted and is presented in Table 4.

The gastrointestinal environment is very different in neonates compared to adults due to the ontogeny of transporters, pH and permeability of the intestinal wall [6,13,163,164,165]. Furthermore, the impact of feeding on drug absorption needs to be considered due to the very different feeding pattern in neonates as well as the co-administration of medicines with foods to improve acceptance and palatability [166,167]. In neonates and infants, attention has been paid to the mixing of medicines with liquid feeds due to the impact on the osmolality of the resulting fluid which may affect GI transit [168,169] although the actual physiological impact is still unclear [170].

Many medicines are mixed with food to improve palatability, yet the impact of this mixing on the absorption of the drug is unknown [171]. The dissolution of drug products in breast and formula milk has been evaluated in vitro to identify whether any effects can be predicted [172]. Other paediatric-relevant dissolution testing systems have also been introduced [173] as well as media that represents the gastric and intestinal fluid from children [163].

Doses used in paediatric populations are often derived from weight-based and surface-area-based dosing equations that are often based on adult data and then scaled based on size and age as an approximation for drug activity in children. However, paediatric growth and development is not a linear process, and there are risks associated with simple scaling to determine doses to use in neonates.

Comprehensive physiologically based pharmacokinetic modelling (PBPK) systems have been introduced that replicate the known parameters of the paediatric GI tract to better predict oral dosing [174,175,176,177,178,179,180,181,182]. However, the major limitation in both in vitro and in silico models is the lack of accurate knowledge about the neonatal and infant intestinal parameters [178,183,184]. These models have focused on oral absorption to date, and there is a need to develop appropriate tools for other routes of drug administration in neonates.

Clinical examples of differences in absorption in neonates compared to adults are scarce. Recent work suggested that the processes underlying changes in oral drug absorption rate typically reach adult levels within one week of birth [35].

### 2.6. Regulatory Challenges 

Useful background information on the European Paediatric Regulation, together with progress reports, is published by the European Commission [7,185]. The US FDA has a similar regulatory framework and many other countries model their approach to the assessment of products on these two sets of regulations [186]. The clarity that these regulations and associated guidelines have provided has advanced the number of new medicines that are available for children. However, the 2017 review of 10 years of the implementation of the European regulations shows there is still a long way to go [185].

Many of the medicines used for neonates have only been developed and authorised for adults and older children. That is, they are used off-label. They may sometimes have been studied extensively perhaps ‘in-house’, but information has often been gathered and published by academia and never submitted by the pharmaceutical industry to regulatory scrutiny [187,188]. Frequently the dosage form that is authorised is not acceptable for neonates or younger children and must be modified for administration via an extemporaneous preparation by the pharmacist or manipulation at the point of administration such as splitting or crushing a solid oral dosage form [189]. Modification of the dosage form may generate an ‘unlicensed’ medicine with attendant uncertainty and risks. Although there is a strong drive to develop licensed medicines for all paediatric subpopulations, as noted throughout this paper this can be extremely challenging for neonates and in some cases industry verified manipulations can provide some increased level of assurance of suitability for use in the interim before a suitable neonate formulation can be licensed [188].

Whilst regulation demands a paediatric investigation plan (PIP) for all new medicines, this does not apply to long-established molecules. There are incentives to study and seek authorisation for age-appropriate dosage forms of off-patent medicines used by children (the Paediatric-Use Marketing Authorisation (PUMA) [190]), but it has not been considered successful in providing the medicines required. The incentives do not appear to be enough to balance the cost of development studies, and despite the acknowledged benefits of using a licensed product, the inevitable cost of such medicines can limit market entry. Enforcement of the use of licensed medicines appears not to be systematic and unlicensed medicines continue to be used.

Since it appears that extemporaneous modification is likely to persist the European Directorate for the Quality of Medicines (EDQM) is establishing a formulary of paediatric medicines prepared extemporaneously. As of January 2019, two monographs are available for public consultation. This seeks to improve access to medicines of demonstrable quality when suitable marketed products are not available [191].

## 3. Burden of Proof

Given all the product, environmental, biopharmaceutics, dose delivery and device factors discussed above, along with the multiplicity of different practices in the clinical setting based upon the age of the neonate, their stage of development and their clinical need, the potential number of pharmaceutical development studies and in particular the compatibility and stability studies are huge. There is a complex matrix of confounding factors such as polypharmacy/coadministrations, low or high concentrations, long residence time and high risk of adsorption to the medical devices used for the administration, e.g., tubing, extractables and leachables assessment, interaction with packaging and unique environmental conditions many of which either alone or in combination can lead to unfavourable clinical outcomes.

The principal aim of a formulator is to design a formulation that provides the intended efficacy for the target patients, ensuring a safe and robust quality profile for the final drug product. However, it would perhaps be unrealistic to test every possible combination. Although this is part of current practices of drug product development, it is important in the context of neonates to reinstate that product developers provide evidence and advice that will cover the majority of anticipated use cases covering both what is acceptable and what is contraindicated from a product quality, safety and efficacy point of view. A lot of this advice can be extrapolated from studies performed for other paediatric subpopulations or adults. However, specific pharmaceutical development studies taking into account the specific requirements of neonates may well be required.

If clinical practice demands that manipulation of formulations or co-dosing etc. that is outside the range of studies performed by the product developer or reported and justified in the literature, then it is the responsibility of the team treating that patient to consider the wisdom of the proposed action. Clearly the pharmacist supporting that team can be of significant importance in evaluating the product quality aspects. The level of practical work supporting those decisions will depend on the circumstances. Thus a ‘one off’ intervention may perhaps be supported on a risk/benefit judgement based on general medical and pharmaceutical principles. If the intervention is used more frequently, then the pharmacy should undertake and publish appropriate compatibility and stability studies. If the intervention is to become commonplace, then formal studies perhaps in association with the original product developer and/or academia will be warranted.

It is essential to assign to appropriate shelf life and/or in-use period for the final product under specific storage conditions (i.e., temperature, humidity and light). Conditions in the NICU may, in fact, represent the best case-controlled environment for the delivery of medications to neonate and drug formulators should investigate other environments in which uncontrolled environmental conditions may present challenges not seen in the NICU. It is vital to demonstrate the in-use usability of the entire product including storage requirements in the likely use environment, access to the packaging, reconstitution procedure, dose measurement, suitability and ease of use of any device required, clarity of instructions. All these factors can/should be assessed in a simulated use evaluation supervised by clinical and/or pharmacy staff using a representative sample of professional staff (i.e., nurse, physician, pharmacy technicians) and where relevant non-professional carers such as parents and guardians. With the increased involvement of staff and carers in the treatment of neonates, evaluation of the role of human factors could be particularly relevant during development.

Studies should aim to mirror clinical practice where possible bearing in mind that the needs for each neonate could vary significantly and might require customized treatment regimens, especially as new treatments become available. Usability study should also include the stability of the formulation over the extended “worst case” time periods being cognisant of regulatory policies on microbiological stability. Limited venous access in neonates can in clinical practice result in little choice but to co-administer drugs. Formulators should be aware of this potential and should extend stability studies to examine Y-site stability of co-administered drugs and also TPN [109,157].

## 4. Conclusions

Development of medicines is challenging per se, and this is even more relevant in the development of medicines for children, let alone preterm babies and neonates. When those children have such rapidly changing physiology and biopharmaceutical characteristics accompanied by critical clinical conditions and requirements, such as is the case for neonates (and in particular preterm neonates), then product development is very challenging. Neonates are still ‘therapeutic orphans’ in terms of access to appropriate drugs and formulations that have been studied and approved by regulators. To design an appropriate formulation for neonates, it is important to understand their physiological status, development and care environment as well as methods of drug administration and the limitations these factors place on formulation development. Given that neonates will usually form a very small fraction of the population that might benefit from a drug, there may be other constraints that limit the ability to provide unique neonatal formulations. A good understanding of the various constraints will allow the formulator to provide for neonates whilst having due regard for the needs of the older population. If the neonate is considered early in the formulation design process, some delays in clinical trials in this population may be avoided.

In recent years, great strides have been made in understanding physiological development in paediatrics in general and neonates in particular. Nonetheless, it is clear that as yet some fundamental information is not available to inform the pharmaceutical development process. For example, further research is needed on the safety of excipients in this population, the development of the CNS and in particular the effect of API and excipients on the maturation of the BBB, the robustness of the skin barrier. The maturation of drug elimination processes and the effect of a rapidly changing fat/lean ratio on drug distribution. Guidance is also needed on compatibility studies that are required and/or desirable in the light of the polypharmacy that is common in the treatment of neonates. Finally, the place of oral therapy with minitablets needs clarification. Further research in these areas will help inform even better product development for this patient group.

Nevertheless, this review has provided information, advice and guidance on factors for the product developer and, in particular, the formulator when seeking to meet the requirements of this highly vulnerable patient group. In general, guidance is given for what is required for treatment in ICU settings but where possible, we have set that in the context of paediatric product development as a whole.

## Figures and Tables

**Table 1:** Characteristic of available types of the vascular catheters (adapted from [21,22,24,25,26].

*Type of Catheter*	*Characteristics*	*Issues*
*Peripheral Venous Catheters*		
*Peripheral venous catheter*	Application: Most IV drugs, isotonic IV fluids, blood transfusions Low flow rates Physicochemical irritation with some drugs results in phlebitis Dwell time: Most need to be removed within three days due to complications	Difficult to insert in the neonates due to the small and hard to visualize vessels
*Central Venous Catheters (CVC)*		
*Umbilical venous catheter (UVC)*	Application: For diagnostic and therapeutic purposes—infusion of medication, TPN, hypertonic IV fluids, central venous pressure and venous blood gas monitoring, blood transfusions Dwell time: Up to 14 days	Suitable for neonates only as the umbilical vein remains for up to two weeks after birth UVC usually inserted within 12 hours of birth if indicated, for parenteral nutrition and/or inotropic support.
*Peripherally inserted central catheter (PICC)*	Application: Medication and IV fluid administration, TPN, blood sampling Suitable for irritant drugs Not suitable for large volume administration in emergency situations (for 28G 20 cm long catheter the max flow is 1 mL/min) Available multi-lumen catheters Made of polyurethane or silicone	Links the benefits of peripheral and central catheter PICC inserted at any time and used for all drugs (in conjunction with UVC helps reduce risk of drug incompatibilities).

**Table 2:** Factors to consider in neonatal parenteral drug formulation and administration.

Chemical and physical compatibility of drug formulation used in multi-drug administration [28] including generic brands
Chemical and physical compatibility of drug formulation used in combination with neonatal TPN [108,109]
Compatibility of drug with diluents typically used in the NICU and stability after dilution
Compatibility of drug formulation while mixing at Y-site junction at different mixing ratios [108,109]
Stability of drug formulation over extended period of time (e.g., over 24 h infusion)
Stability of drug formulation exposed to different environmental conditions (high temperature, strong light, high oxygen levels) [110]
Stability and compatibility of excipients used in drug formulation
Stability and compatibility of excipients used in drug formulation with IV administration set and container
Compatibility of drug formulation with IV administration set and container
Strength(s)/concentration of drug that can cover neonatal weight- or age-bands as well as fluid restricted patients
Performance of medical equipment delivering drug—volumetric and smart pumps, syringe drivers
Design of IV administration set minimising drug delivery delays

**Table 3:** Assessment of drug product leachables associated with pharmaceutical packaging/delivery systems (modified from “FDA/CDER/CBER risk-based approach to consideration of leachables” (USP—General chapter <1664>) [148]).

Degree of Concern Associated with the Route of Administration	Likelihood of Packaging Component–Dosage Form Interaction		
	High	Medium	Low
Highest	Inhalation aerosol and spray	Injections and injectable suspensions, inhalation solution	Sterile powders and powders for injection, inhalation powders
High	Transdermal ointment and patches	Ophthalmic solutions and suspension, nasal aerosol and spray	
Low	Topic solutions and suspensions, topical and lingual aerosol, oral solutions and suspensions		Oral tablets and oral (hard and soft gelatin) capsules, topical powders, oral powders

**Table 4:** Summary of differences between neonatal and adult physiology that affect absorption/distribution of drugs (extracted from [13]).

Route of Administration	Impact on Absorption/Distribution	Reasons
Oral	Altered absorption	Neonatal pH is elevated in the stomach (increased for basic drugs and reduced for acidic drugs) Immature ontogeny of transporter expression
	Reduced absorption	Slower gastric emptying Reduced relative surface area in the intestine
	Increased absorption	Slower intestinal transit Reduced intestinal P-glycoprotein expression
Rectal	Decreased surface area	Reduced relative surface area of rectum
Respiratory	Decreased absorption	Immature lung branching and development Reduced lung capacity and inspiratory flow
Nasal	No data shown	Potential for irritation in the nasal mucosa in neonates
Dermal and transdermal	Increased absorption	Higher BSA/kg ratio Thinner stratum cornea layer More hydrated stratum corneum Higher relative surface area to bodyweight
IV	Reduced distribution	Reduced blood volume
Intramuscular	Reduced distribution	Reduced muscle mass
	Altered distribution	Variable muscle blood flow
Subcutaneous	Reduced distribution	Reduced subcutaneous fat
